# Clinical Characteristics and Short-Term Outcomes of Severe Patients With COVID-19 in Wuhan, China

**DOI:** 10.3389/fmed.2020.00491

**Published:** 2020-08-06

**Authors:** Xiaobo Feng, Peiyun Li, Liang Ma, Hang Liang, Jie Lei, Wenqiang Li, Kun Wang, Yu Song, Shuai Li, Wei Yang, Cao Yang

**Affiliations:** ^1^Department of Orthopaedics, Union Hospital, Tongji Medical College, Huazhong University of Science and Technology, Wuhan, China; ^2^Department of Clinical Nutrition, Wuhan Children's Hospital (Wuhan Maternal and Child Healthcare Hospital), Tongji Medical College, Huazhong University of Science and Technology, Wuhan, China; ^3^Hubei Key Laboratory of Food Nutrition and Safety, Department of Nutrition and Food Hygiene, Tongji Medical College, Huazhong University of Science and Technology, Wuhan, China; ^4^Department of Nutrition and Food Hygiene and MOE Key Lab of Environment and Health, School of Public Health, Tongji Medical College, Huazhong University of Science and Technology, Wuhan, China

**Keywords:** COVID-19, SARS-CoV-2, severe patients, short-term outcomes, inflammation

## Abstract

**Background:** A novel pneumonia (COVID-19) spread rapidly throughout worldwide, in December, 2019. Most of the deaths have occurred in severe and critical cases, but information on prognostic risk factors for severely ill patients is incomplete. Further research is urgently needed to guide clinicians, and we therefore prospectively evaluate the clinical outcomes of 114 severely ill patients with COVID-19 for short-term at the Union Hospital in Wuhan, China.

**Methods:** In this single-centered, prospective, and observational study, we enrolled 114 severely ill patients with confirmed COVID-19 from Jan 23, 2020, to February 22, 2020. Epidemiological, demographic, laboratory, treatment, and outcome data were recorded, and the risk factors for poor outcome were analyzed.

**Results:** Among the 114 enrolled patients with a mean age of 63.96 ± 13.41 years, 94 (82.5%) patients were classified as a good outcome group. Common clinical manifestations included fever, cough, and fatigue. Compared with the good outcome group, 20 (17.5%) patients in the poor outcome group more frequently exhibited lymphopenia, and lower levels of albumin, partial arterial oxygen pressure, higher levels of lactate dehydrogenase, creatine kinase, hypersensitive troponin I, C-reactive protein, ferritin, blood urea nitrogen, and D-dimer, as well as markedly higher levels of IL-6 and IL-10. Absolute numbers of T lymphocytes, CD8 + T cells, decreased in almost all the patients and were markedly lower in the poor outcome group than the good outcome group. We also found that traditional Chinese medicine can significantly improve the patient's condition, which is conducive to the transformation from a severe to mild condition. In addition, univariate and multivariate Cox analyses of potential factors for poor outcome patients indicated that cytokine storms and uncontrolled inflammation responses as well as liver, kidney, and cardiac dysfunction are related to the development of a poor outcome.

**Conclusion:** In summary, we reported this single-centered, prospective, and observational study for short-term outcome in severe patients with COVID-19. We found that cytokine storms and uncontrolled inflammation responses as well as liver, kidney, and cardiac dysfunction may play important roles in the final outcome of severely ill patients with COVID-19. Our study will allow clinicians to benefit and rapidly estimate the likelihood of a short-term poor outcome for severely ill patients.

## Introduction

A pneumonia caused by a novel coronavirus, severe acute respiratory syndrome corona virus 2 (SARS-CoV-2), spread rapidly throughout worldwide in December 2019 (1). Despite progress made in our understanding of the characteristics of the disease, there are currently no drugs to combat SARS-CoV-2, and patients are primarily provided with supportive treatment. Several studies have indicated that the main symptoms of coronavirus disease 2019 (COVID-19) include fever, cough, and dyspnea (2–5). Huang et al. described the epidemiological, clinical, laboratory, and radiological characteristics of COVID-19, as well as various treatment strategies and outcomes, among 41 patients during the first wave of hospitalizations. They also compared clinical characteristics between patients treated in an intensive care unit (ICU) and those treated in non-ICU (2). Yang et al. also performed a detailed analysis of the patients critically ill with SARS-CoV-2 infection (5). In an analysis of 74 patients with COVID-19 exhibiting gastrointestinal symptoms, Jin et al. suggested that non-classical symptoms have been overlooked, posing a threat to the public (6). Wu et al. further noted that the risk of acute respiratory distress syndrome (ARDS) and death is increased in older adults (≥65 years old) with COVID-19 (7). Guo et al. found that diabetes is a risk factor for patients with COVID-19 (8). However, few prospective studies have explored the short-term outcomes of severely ill patients under current medical treatment and the risk factors that affect the short-term outcomes of severely ill patients, especially pneumonia patients with certain chronic diseases, which accounted for the majority of deaths. Here, we used a single-centered, prospective method to describe the basic clinical characteristics and short-term outcomes of severe patients in Union hospital, Wuhan, and we further aimed to explore the potential risk factors for poor outcomes among these patients using Cox proportional hazard models.

## Methods

### Study Design and Participants

This single-center, prospective study included 114 severe patients with confirmed COVID-19 pneumonia hospitalized at the Union Hospital in Wuhan, China, which is a hospital designated to treat patients with COVID-19. From January 23, 2020, to February 22, 2020, we continuously enrolled patients diagnosed with COVID-19 based on interim guidance provided by the World Health Organization (WHO). Based on the Diagnosis and Treatment Scheme for SARS- CoV-2 of Chinese (The Seven Edition), severe patients were diagnosed if one or more of following criteria were met: dyspnea with respiratory rate (RR) ≥30 times/min, resting finger oxygen saturation ≤93%, and artery PaO_2_/FiO_2_ ≤300 mm Hg (1 mm Hg = 0.133 kPa). This study was approved by the Ethics Commission of Wuhan Union Hospital of Tongji Medical College, Huazhong University of Science and Technology. Written informed consent was waived due to the emergency of this infectious diseases.

### Data Collection

Data related to clinical characteristics were collected using a case record form modified from the standardized International Severe Acute Respiratory and Emerging Infection Consortium case report form. Epidemiological and demographic data, including age, sex, and coexisting disorders, were also collected. The Baseline laboratory indices and radiographic findings were obtained from clinical electronic medical records. Moreover, the treatment strategies and outcomes were collected until the day of death /discharge or for the first 28 days after a diagnosis of severe illness, whichever was shorter. All missing or vague data, were obtained by communicating with patients and their families. All data were checked by two physicians (Xiaobo Feng and Liang Ma), and a third researcher (Wei Yang) adjudicated any difference in interpretation between the two primary reviewers.

### Outcomes

Clinical outcomes after 28 days of consecutive observations were divided into two categories. Patients that had been discharged, those whose condition had been deemed non-severe, and those not requiring mechanical ventilation were considered to have experienced good outcomes. Patients requiring mechanical ventilation and those who had died were considered to have experienced poor outcomes. The criteria for discharge were as follows: normal temperature for more than 3 days (T < 37.3°C), significant improvement in respiratory symptoms, pulmonary imaging showing significant improvement in acute exudative lesions, and nucleic acid tests negative for respiratory tract specimens such as sputum and nasopharyngeal swabs for two consecutive samplings (at least 24 h after sampling). Patients with mild clinical symptoms and no signs of pneumonia on radiography were considered to be non-severe. ARDS and shock were confirmed by the WHO guidance for COVID-19. Acute kidney injury was defined according to the serum creatinine. Cardiac injury was identified by the serum concentration of hypersensitive cardiac troponin I (hsTNI) and, if it was above the upper limit of the reference range (>28 pg/mL), measured in the laboratory of Union Hospital (5).

### Statistics

Continuous variables were expressed as means ± SDs if normally distributed and medians (IQRs) if skewed distributed while categorical variables were summarized as number (%). Differences between the characteristics of outcome groups were assessed using students *t*-test or Mann-Whitney U-test for continuous variables and chi-square tests for categorical variables. In addition, univariate and multivariate Cox proportional hazard models were used to determine hazard ratios (HRs) and 95% confidence intervals (CIs) of poor outcome in severe patients with COVID-19. The candidate risk factors included demographic and epidemiological characteristics as well as some laboratory indices. We determined the cut points of levels according to normal range, actual distribution, and clinical significance of each index. Adjustments were made for potential confounders, including age and sex. For risk factors identified in Cox analyses, we used restricted cubic spline model to further explore the potential dose-response relationship between factors and poor outcome risk. The referent (HR = 1) was set according to the cut point in Cox analyses. *P* < 0.05 was considered statistically significant. All data were analyzed using SPSS (23.0 IBM SPSS).

## Results

### Clinical Outcomes

**Figure 1:**
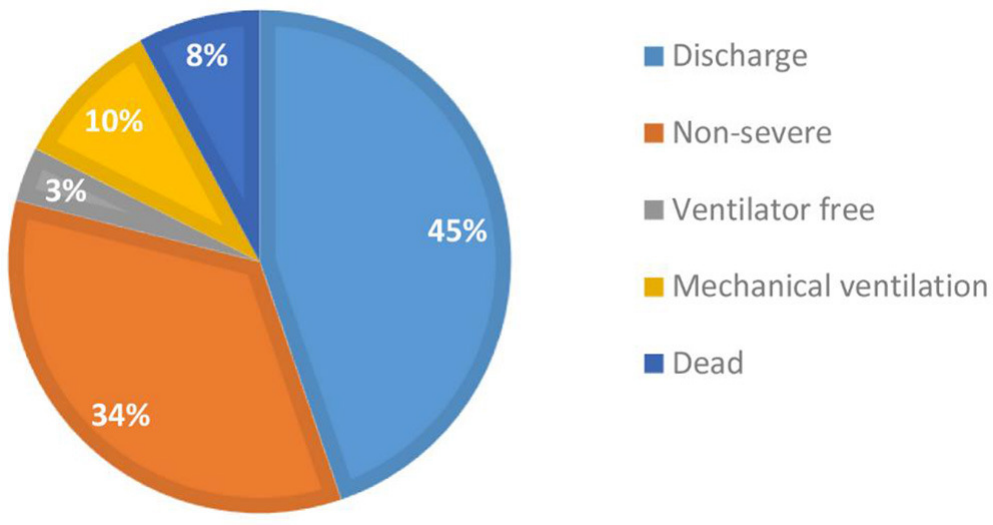
Distribution of short-term (28 days) outcomes of severely ill patients with COVID-19 (*N* = 114).

As of March 21, 2020, a total of 114 patients diagnosed with and treated for severe COVID-19 were enrolled in this study. Twenty-eight days after a diagnosis of severe COVID-19, good and poor outcomes were observed in 94 and 20 patients, respectively. As shown in Figure 1, 51 (45%) patients were alive and had been discharged, 39 (34%) had transitioned to non-severe illness, four (3%) remained severely ill but did not require a ventilator, i.e., severe status and ventilator free, 11 (10%) were alive but remained ventilated, and nine (8%) had died.

### Demographics and Characteristics

The general demographic and epidemiological characteristics of all enrolled patients are summarized in Table 1. The mean age was 63.96 ± 13.41 years, 62 (54.4%) were older than 65 years and 71 (62.3%) were male. A total of 89 (78.1%) severe patients have a chronic medical illness, and the most coexisting disorders were hypertension with 62 (54.4%), diabetes with 39 (34.2%) and cardiovascular disease with 31 (27.2%). No significant differences were observed in such characteristics between outcome groups (*P* ≥ 0.05 for all). Table 2 displays the clinical characteristics of the patients. The duration from onset of symptoms to diagnosis of COVID-19 was 4.0 (2.0–7.0) days, while the duration from the onset of symptoms to the diagnosis of severe illness was 10.0 (6.0–14.3) days. For 114 patients, the most common symptoms at initial diagnosis were fever in 78 (68.4%), cough in 49 (43.0%), chest tightness in 34 (29.8%), and fatigue in 30 (26.3%) patients. Other symptoms, including shortness of breath in 18 (15.8%), anorexia in 12 (10.5%), chill in 12 (10.5%), myalgia in 10 (8.8%), sputum in nine (7.9%), headache in eight (7.0%), diarrhea in eight (7.0%), chest pain in three (2.6%), stomachache in three (2.6%) and nausea in three (2.6%) were relatively rare. As for oxygen saturation, patients in the poor outcome group had significantly lower levels than those in the good outcome group [median (IQR): 91 (90–93%) in the good outcome group and 81 (74–88%) in the bad outcome group, *P* < 0.001].

**Table 1:** Demographic and epidemiological characteristics of severe patients with COVID-19.

	**Total (*N =* 114)**	**Good outcome (*N =* 94)**	**Poor outcome (*N =* 20)**	***P***
Age (years)	63.96 ± 13.41	62.85 ± 13.65	69.15 ± 11.08	0.056
<65	52 (45.6)	46 (48.9)	6 (30.0)	0.123
≥65	62 (54.4)	48 (51.1)	14 (70.0)	
Sex, male	71 (62.3)	58 (61.7)	13 (65.0)	0.782
Hospital infection	7 (6.1)	6 (6.4)	1 (5.0)	>0.999
Coexisting disorders	89 (78.1)	73 (77.7)	16 (80.0)	>0.999
Diabetes	39 (34.2)	34 (36.2)	5 (25.0)	0.339
Hypertension	62 (54.4)	50 (53.2)	12 (60.0)	0.579
Hyperlipidemia	17 (14.9)	15 (16.0)	2 (10.0)	0.739
Cardiovascular diseases	31 (27.2)	24 (25.5)	7 (35.0)	0.388
Cerebrovascular diseases	6 (5.3)	3 (3.2)	3 (15.0)	0.110
Cancer	10 (8.8)	9 (9.6)	1 (5.0)	0.825
Chronic renal diseases	6 (5.3)	4 (4.3)	2 (10.0)	0.622
Chronic liver diseases	4 (3.5)	3 (3.2)	1 (5.0)	0.543
Chronic Obstructive Pulmonary Disease	11 (9.6)	9 (9.6)	2 (10.0)	>0.999
Neuropsychiatric disorders	3 (2.6)	2 (2.1)	1 (5.0)	0.443
History of surgery	33 (28.9)	26 (27.7)	7 (35.0)	0.511

*Values are n (%) for categorical data, means ± SDs for normally distributed data, or medians (IQRs) for non-normally distributed data*.

**Table 2:** Clinical characteristics of severe patients with COVID-19.

	**Total (*N =* 114)**	**Good outcome (*N =* 94)**	**Poor outcome (*N =* 20)**	***P***
**Onset of symptom to, d**				
Diagnosis	4.0 (2.0–7.0)	4.0 (2.0–7.0)	4.5 (2.3–10.8)	0.517
Serious illness	10.0 (6.0–14.3)	10.0 (6.0–15.0)	8.0 (5.0–14.0)	0.540
**Signs and symptoms at initial**				
Fever	78 (68.4)	63 (67.0)	15 (75.0)	0.486
Chest tightness	34 (29.8)	27 (28.7)	7 (35.0)	0.577
Shortness of breath	18 (15.8)	13 (13.8)	5 (25.0)	0.365
Cough	49 (43.0)	39 (41.5)	10 (50.0)	0.485
Sputum	9 (7.9)	8 (8.5)	1 (5.0)	0.943
Fatigue	30 (26.3)	28 (29.8)	2 (10.0)	0.068
Headache	8 (7.0)	7 (7.4)	1 (5.0)	>0.999
Myalgia	10 (8.8)	8 (8.5)	2 (10.0)	>0.999
Chest pain	3 (2.6)	3 (3.2)	0 (0.0)	1.000
Anorexia	12 (10.5)	12 (12.8)	0 (0.0)	0.198
Chill	12 (10.5)	8 (8.5)	4 (20.0)	0.263
Stomachache	3 (2.6)	3 (3.2)	0 (0.0)	>0.999
Diarrhea	8 (7.0)	7 (7.4)	1 (5.0)	>0.999
Nausea	3 (2.6)	2 (2.1)	1 (5.0)	0.443
Temperature at disease onset (°C)	38.1 (36.7–38.8)	38.1 (36.7–38.7)	38.2 (37.0–39.0)	0.561
<37.4	36 (31.6)	31 (33.0)	5 (25.0)	0.622
37.4–39.0	63 (55.3)	50 (53.2)	13 (65.0)	
>39.0	15 (13.2)	13 (13.8)	2 (10.0)	
**Signs and symptoms at hospital admission**				
Fever	32 (28.1)	25 (26.6)	7 (35.0)	0.448
Chest tightness	50 (43.9)	46 (48.9)	4 (20.0)	0.018
Shortness of breath	68 (59.6)	49 (52.1)	19 (95.0)	<0.001
Cough	9 (7.9)	7 (7.4)	2 (10.0)	>0.999
Fatigue	43 (37.7)	37 (39.4)	6 (30.0)	0.433
Headache	6 (5.3)	6 (6.4)	0 (0.0)	0.542
Myalgia	5 (4.4)	5 (5.3)	0 (0.0)	0.585
Chest pain	3 (2.6)	3 (3.2)	0 (0.0)	>0.999
Anorexia	18 (15.8)	16 (17.0)	2 (10.0)	0.657
Diarrhea	10 (8.8)	7 (7.4)	3 (15.0)	0.516
Nausea	2 (1.8)	2 (2.1)	0 (0.0)	>0.999
**General signs at admission**				
Temperature (°C)	36.7 (36.4–37.4)	36.7 (36.4–37.3)	38.2 (37.0–39.0)	0.279
<37.4	85 (74.6)	72 (76.6)	13 (65.0)	0.535
37.4–39.0	26 (22.8)	20 (21.3)	6 (30.0)	
>39.0	3 (2.6)	2 (2.1)	1 (5.0)	
Heart rate (/min)	86 (78–102)	87 (78–102)	88 (78–102)	0.456
<100	83 (72.8)	69 (73.4)	14 (70.0)	0.756
>100	31 (27.2)	25 (26.6)	6 (30.0)	
Respiratory rate (/min)	20 (20–23)	20 (20–22)	21 (20–30)	0.122
<20	22 (19.3)	19 (20.2)	3 (15.0)	0.760
≥20	92 (80.7)	75 (79.8)	17 (85.0)	
Oxygen saturation (%)	90 (88–92)	91 (90–93)	81 (74–88)	<0.001
<90	35 (30.7)	19 (20.2)	16 (80.0)	<0.001
≥90	79 (69.3)	75 (79.8)	4 (20.0)	

*Values are n (%) for categorical data, means ± SDs for normally distributed data, or medians (IQRs) for non-normally distributed data*.

### Laboratory and CT Findings

In terms of the 114 severe patients, many laboratory indicators differed significantly between outcome groups (Table 3). Compared with the good outcome group, the absolute counts of neutrophils 6.25 (4.69–9.20) vs. 3.48 (2.54–5.23), c-reactive protein 102.15 (78.07–122.90) vs. 48.95 (15.08–83.98), D dimer 2.10 (1.22–3.07) vs. 0.96 (0.41–.78), total bilirubin 19.20 (9.25–33.05) vs. 11.05 (8.53–14.05), blood urea nitrogen 9.02 (5.26–11.30) vs. 4.11 (3.11–5.04), creatine kinase 151.50 (50.50–218.50) vs. 62.00 (46.75–110.50), lactate dehydrogenase 638.00 (436.00–923.00) vs. 259.50 (213.75–382.50), hypersensitive cardiac troponin I 60.70 (18.48–298.98) vs. 4.10 (1.70–10.83), ferritin 679.00 (573.90–993.15) vs. 321.80 (231.00–532.88), interleukin-6 (IL-6) 76.10 (19.05–192.88) vs. 21.23 (7.23–47.61), and IL-10 6.59 (4.58–11.78) vs. 4.64 (3.65–6.18) were significantly higher in poor outcome. Besides, total protein 60.40 (56.78–64.05) vs. 63.80 (59.33–68.50) and PaO2 68.15 (49.00–77.75) vs. 81.00 (74.75–89.00) were significantly lower in poor outcome group. For chest X- ray/CT, 107 (93.9) patients had Ground-glass opacity. These data indicated that the uncontrolled inflammation responses, infection, liver, and kidney dysfunction, and hypoxia may contribute to poor outcomes in patients with COVID-19.

**Table 3:** Laboratory and radiographic findings at baseline of severe patients with COVID-19.

	**Total (*N =* 114)**	**Good outcome (*N =* 94)**	**Poor outcome (*N =* 20)**	***P***
**Hematologic**				
Leucocytes (×10^9^ /L)	6.28 ± 3.36	5.74 ± 2.49	8.78 ± 5.36	0.022
Neutrophils (×10^9^ /L)	3.88 (2.65–5.81)	3.48 (2.54–5.23)	6.25 (4.69–9.20)	<0.001
Lymphocytes (×10^9^ /L)	0.87 (0.65–1.31)	0.92 (0.70–1.43)	0.67 (0.43–0.89)	0.001
Monocytes (×10^9^ /L)	0.44 (0.30–0.59)	0.48 (0.33–0.61)	0.34 (0.22–0.40)	0.009
Platelets (×10^9^ /L)	192.00 (140.74–269.75)	205.00 (142.75–272.50)	165.00 (138.25–218.00)	0.160
Hemoglobin (g/L)	122.04 ± 17.78	122.65 ± 16.75	119.15 ± 22.29	0.427
CD4+ T cells (%)	43.54 (36.45–53.41)	44.98 (36.53–53.20)	39.95 (31.46–57.32)	0.687
CD8+ T cells (%)	21.53 (16.48–28.55)	22.11 (17.61–29.95)	17.90 (14.00–23.45)	0.025
**Coagulation function**				
Prothrombin time (s)	13.5 (12.8–14.3)	13.40 (12.70–14.23)	14.25 (12.93–15.28)	0.072
Activated partial thromboplastin time (s)	38.00 (35.18–41.53)	38.00 (35.10–41.43)	38.45 (35.45–43.98)	0.398
Fibrinogen (g/L)	4.98 ± 1.48	5.05 ± 1.43	4.66 ± 1.67	0.282
Thrombin time (s)	17.35 (16.58–18.30)	17.35 (16.77–18.15)	17.20 (15.35–19.18)	0.467
D-dimer (mg/L)	1.06 (0.51–2.10)	0.96 (0.41–1.78)	2.10 (1.22–3.07)	0.005
**Biochemical**				
Alanine aminotransferase (U/L)	45.50 (26.00–74.25)	44.50 (25.75–72.50)	49.00 (29.25–80.25)	0.427
Aspartate aminotransferase (U/L)	39.50 (26.75–64.50)	38.50 (26.00–57.25)	44.00 (29.50–114.75)	0.197
Total bilirubin (μmol/L)	11.35 (8.93–16.15)	11.05 (8.53–14.05)	19.20 (9.25–33.05)	0.024
Total protein (g/L)	63.10 (58.48–67.70)	63.80 (59.33–68.50)	60.40 (56.78–64.05)	0.015
Albumin (g/L)	34.65 (30.350–38.60)	35.80 (31.85–39.10)	30.05 (27.10–32.65)	<0.001
Globulin (g/L)	29.15 (25.70–31.65)	28.95 (25.68–30.78)	30.35 (29.63–35.50)	0.057
Prealbumin (mg/L)	120 (90–153)	120 (90–151)	93 (82–181)	0.145
Blood urea nitrogen (mmol/L)	4.39 (3.27–6.13)	4.11 (3.11–5.04)	9.02 (5.26–11.30)	<0.001
Creatinine (μmol/L)	74.35 (60.00–87.85)	74.35 (60.28–86.38)	71.80 (53.70–95.60)	0.636
Creatine kinase (U/L)	66.50 (46.75–133.50)	62.00 (46.75–110.50)	151.50 (50.50–218.50)	0.046
Creatine kinase-MB (U/L)	17.00 (14.00–23.00)	17.00 (14.00–22.00)	19.50 (13.00–31.75)	0.234
Lactate dehydrogenase (U/L)	286.00 (223.75–452.25)	259.50 (213.75–382.50)	638.00 (436.00–923.00)	<0.001
Hypersensitive cardiac troponin I (ng/L)	5.70 (2.10–19.05)	4.10 (1.70–10.83)	60.70 (18.48–298.98)	<0.001
Glucose (mmol/L)	6.20 (5.20–8.31)	6.13 (5.16–7.63)	8.26 (5.81–13.42)	0.013
Serum potassium (mmol/L)	4.03 (3.70–4.50)	4.07 (3.72–4.51)	3.97 (3.59–4.36)	0.344
Serum sodium (mmol/L)	138.85 (136.30–142.00)	138.20 (136.00–141.68)	142.40 (138.85–146.93)	0.001
Serum calcium (mmol/L)	2.15 ± 0.18	2.17 ± 0.16	2.04 ± 0.20	0.003
Serum phosphorus (mmol/L)	1.00 (0.88–1.11)	1.00 (0.89–1.11)	0.95 (0.73–1.12)	0.381
Serum chlorine (mmol/L)	100.48 ± 5.10	100.03 ± 4.45	102.61 ± 7.20	0.137
Lactate concentration (mmol/L)	1.60 (1.38–1.80)	1.50 (1.30–1.70)	2.10 (1.70–2.40)	<0.001
Positive Urinary protein, n	47 (41.2)	33 (35.1)	14 (70.0)	0.004
Positive Urinary glucose, n	8 (7.0)	7 (7.4)	1 (5.0)	>0.999
Positive urinary occult blood, n	31 (27.2)	21 (22.3)	10 (50.0)	0.012
**Blood gas characteristics**				
pH	7.39 (7.36–7.42)	7.38 (7.36–7.42)	7.41 (7.31–7.46)	0.636
Arterial partial pressure of oxygen (mm Hg)	79.00 (70.00–88.00)	81.00 (74.75–89.00)	68.15 (49.00–77.75)	<0.001
Arterial partial pressure of carbon dioxide (mm Hg)	43.00 (38.00–46.00)	42.80 (38.00–45.00)	45.50 (35.60–57.00)	0.198
**Infection-related biomarkers**				
Interleukin 2 (pg/mL)	2.70 (2.43–3.04)	2.70 (2.47–3.02)	2.69 (2.41–3.60)	0.991
Interleukin 4 (pg/mL)	2.16 (1.85–2.60)	2.16 (1.89–2.52)	2.21 (1.68–3.50)	0.729
Interleukin 6 (pg/mL)	23.28 (8.31–54.23)	21.23 (7.23–47.61)	76.10 (19.05–192.88)	0.002
Interleukin 10 (pg/mL)	4.91 (3.92–6.74)	4.64 (3.65–6.18)	6.59 (4.58–11.78)	0.001
Tumor necrosis factor-α (pg/mL)	2.63 (2.11–4.80)	2.63 (2.11–4.80)	2.67 (2.07–4.66)	0.838
Interferon-γ (pg/mL)	2.50 (1.96–3.20)	2.46 (1.96–3.20)	2.58 (1.89–3.49)	0.571
C-reactive protein (mg/L)	67.95 (20.50–103.25)	48.95 (15.08–83.98)	102.15 (78.07–122.90)	<0.001
Ferritin (ng/mL)	390.60 (261.70–721.00)	321.80 (231.00–532.88)	679.00 (573.90–993.15)	0.001
IgM, *n*	60 (68.2)	52 (69.3)	8 (61.5)	0.748
IgG, *n*	88 (100.0)	75 (100.0)	13 (100.0)	NA
**Chest X- ray/CT findings**				
Ground-glass opacity	107 (93.9)	87 (92.6)	20 (100.0)	0.351
Unilateral pneumonia	11 (9.6)	11 (11.7)	0 (0.0)	0.208
Bilateral pneumonia	102 (89.5)	82 (87.2)	20 (100.0)	0.122
Interstitial abnormalities	21 (18.4)	15 (16.0)	6 (30.0)	0.200

*Data are n (%), means ± SDs or medians (IQRs) when appropriate*.

### Complications and Treatments

As shown in Table 4, patients with severe COVID-19 had complications including acute liver injury, ARDS, acute kidney injury, arrhythmia, acute myocardial injury, Disseminated Intravascular Coagulation (DIC), rhabdomyolysis, and septic shock. However, no patients in the good outcome group experienced septic shock. All patients in the poor outcome group experienced ARDS. Acute myocardial injury, acute kidney injury, arrhythmia, rhabdomyolysis, and DIC were significantly higher than their counterparts in 13.8, 22.3, 17.0, 2.1, and 2.1% of patients with COVID-19 in the good outcome group, respectively. All 114 patients with severe COVID-19 were treated with antibiotics and high flow nasal cannula, while 25 (21.9%) were treated with non-invasive mechanical ventilation and 22 (19.3%) with invasive mechanical ventilation treatment. Six (5.3%) patients were treated with extracorporeal membrane oxygenation (ECMO) and all of whom were in the poor outcome group. Almost all [113 (99.1%)] patients received antiviral treatment, including arbidol hydrochloride capsules (0.2 g three times daily), lopinavir, and ribavirin (500 mg two times daily) via the oral route. Furthermore, as many as 41.2% patients received glucocorticoid therapy. Sixty-four (56.1%) patients received immunoglobulin treatment, and 49 (43.0%) patients were treated with parenteral nutrition; the percentage was higher in the poor outcome group than in the good outcome group [20 (100.0%) vs. 29 (30.9%)]. Two patients (1.8%) were treated with renal replacement therapy and 20 (17.5%) with vasoconstrictive agents, and it was higher than in the good outcome group [19 (95.0%) vs. 1 (1.1%)]. Moreover, the patients were given Traditional Chinese medicine (TCM) based on the protocol (9). All 20 patients in the poor group were transferred to the ICU, which was significantly higher than that of 9 (9.6%) in the good outcome group.

**Table 4:** Complications and treatments of severe patients with COVID-19.

	**Total (*N =* 114)**	**Good outcome (*N =* 94)**	**Poor outcome (*N =* 20)**	***P***
**Complications**				
Shock	8 (7.0)	0 (0.0)	8 (40.0)	<0.001
Acute respiratory distress syndrome	41 (36.0)	21 (22.3)	20 (100.0)	<0.001
Acute renal injury	35 (30.7)	21 (22.3)	14 (70.0)	<0.001
Acute myocardial injury	28 (24.6)	13 (13.8)	15 (75.0)	<0.001
Acute liver function injury	69 (60.5)	57 (60.6)	12 (60.0)	0.958
Arrhythmia	31 (27.2)	16 (17.0)	15 (75.0)	<0.001
Rhabdomyolysis	11 (9.6)	2 (2.1)	9 (45.0)	<0.001
Disseminated intravascular coagulation	15 (13.2)	2 (2.1)	13 (65.0)	<0.001
**Treatment**				
Antibiotic treatment	114 (100.0)	94 (100.0)	20 (100.0)	NA
Anticoronavirus treatment	113 (99.1)	93 (98.9)	20 (100.0)	>0.999
Glucocorticoids	47 (41.2)	28 (29.8)	19 (95.0)	<0.001
Oxygen therapy	114 (100.0)	94 (100.0)	20 (100.0)	NA
Immunoglobulin	64 (56.1)	45 (47.9)	19 (95.0)	<0.001
Parenteral nutrition	49 (43.0)	29 (30.9)	20 (100.0)	<0.001
Admission to intensive care unit	29 (25.4)	9 (9.6)	20 (100.0)	<0.001
Non-invasive ventilation	25 (21.9)	13 (13.8)	12 (60.0)	<0.001
Invasive mechanical ventilation	22 (19.3)	4 (4.3)	18 (90.0)	<0.001
Extracorporeal membrane oxygenation	6 (5.3)	0 (0.0)	6 (30.0)	<0.001
Vasoconstrictive agents	20 (17.5)	1 (1.1)	19 (95.0)	<0.001
Renal replacement therapy	2 (1.8)	0 (0.0)	2 (10.0)	0.029
Traditional Chinese medicine	86 (75.4)	77 (81.9)	9 (45.0)	0.001
Trastuzumab	13 (11.4)	12 (12.8)	1 (5.0)	0.459
Infusions of blood plasma	4 (3.5)	2 (2.1)	2 (10.0)	0.141
Onset of severe illness to, d^*^				
1st for RT-PCR (–)	14.0 (11.0–18.0)	14.0 (10.0–18.0)	19.0 (15.5–24.0)	<0.001
2nd for RT-PCR (–)	17.5 (14.0–22.0)	17.0 (14.0–21.5)	24.0 (22.0–27.5)	<0.001

*Data are n (%) or medians (IQRs) when appropriate*.

* *Data available for 98 patients*.

### Prediction of Risk Factors for Severe COVID-19 in the Poor Outcome Group

**Figure 2:**
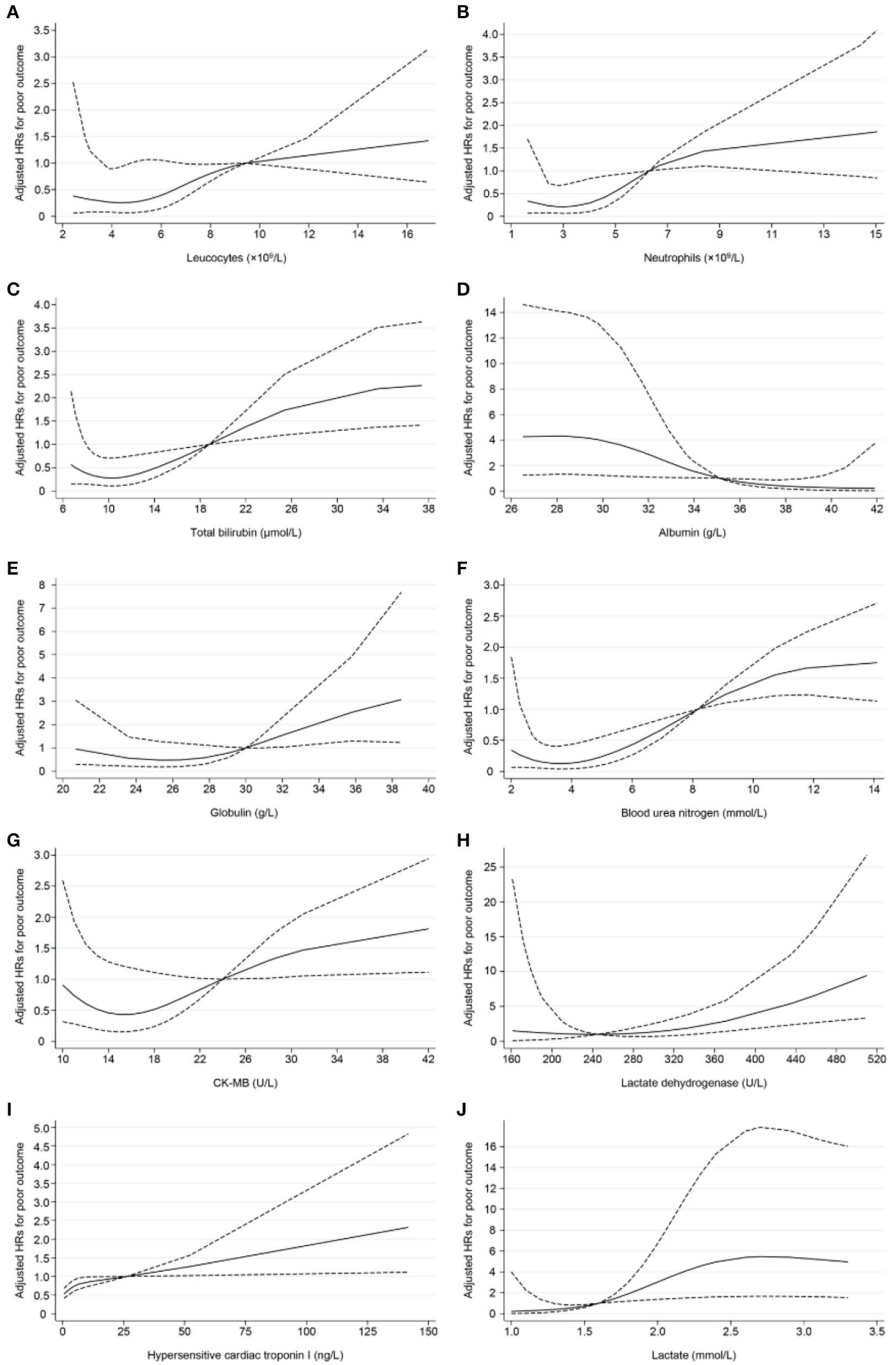
Non-linear dose–response relationship between 10 indices and poor outcome risk. Hazard ratios (HRs) were adjusted for age and gender. Dotted lines represent the 95% CIs for the fitted trend. **(A)** Leucocytes (×10^9^ /L), referent (HR = 1): 9.5; **(B)** Neutrophils (×10^9^ /L), referent: 6.3; **(C)** Total bilirubin (μmol/L), referent: 19.0; **(D)** Albumin, referent: 35.0 (g/L); **(E)** Globulin (g/L), referent: 30.0; **(F)** Blood urea nitrogen (mmol/L), referent: 8.2; **(G)** CK-MB (U/L), referent: 24.0; **(H)** Lactate dehydrogenase (U/L), referent: 245; **(I)** Hypersensitive cardiac troponin I (ng/L), referent: 26.2; **(J)** Lactate (mmol/L), referent: 1.6.

Tables 5, 6 display the results of univariate and multivariate Cox analyses of potential risk factors for short-term outcomes in severe patients with COVID-19. Our results indicated that, for severe patients, higher levels of oxygen saturation (HR, 0.123; 95% CI, 0.041–0.369), albumin (HR, 0.060; 95% CI, 0.008–0.460), and arterial partial pressure of oxygen (HR, 0.321; 95% CI, 0.106–0.973) were associated with decreased risk of developing poor outcome within 28 days. In the other hand, higher levels of leucocytes (HR, 5.575; 95% CI, 2.080–14.943), neutrophils (HR, 2.566; 95% CI, 1.022–6.443), total bilirubin (HR, 6.171; 95% CI, 2.458–15.496), globulin (HR, 2.526; 95% CI, 1.027–6.211), blood urea nitrogen (HR, 5.640; 95% CI, 2.193–14.509), creatine kinase-MB (HR, 3.032; 95% CI, 1.203–7.644), lactate dehydrogenase (HR, 4.607; 95% CI, 1.057–20.090), hypersensitive cardiac troponin I (HR, 5.023; 95% CI, 1.921–13.136), lactate concentration (HR,15.721; 95% CI, 2.099–117.777), Interleukin-10 (HR, 3.551; 95% CI, 1.280–9.857), and C-reactive protein (HR, 5.275; 95% CI, 1.517–18.344) were associated with increased risk of poor outcome development. For all the factors analyzed above, increased concentration of lactate (≥1.6 mmol/L) and total bilirubin (≥19.0 μmol/L) might be the most important predictors of poor outcome in the early stage. As shown in Figure 2, non-linear dose-response relationship was also found between 10 indices and poor outcome risk in the cubic spline model.

**Table 5:** Univariate and multivariate analyses of potential factors (demographic and epidemiologic) predicting poor outcome.

**Factors**	**Level**	**Crude HR (95% CI)**	***P***	Adjusted HR (95% CI)^*^	***P***
Age (years)	≥65 vs. <65	2.192 (0.842–5.708)	0.108	2.184 (0.839–5.687)	0.110
Sex	Female vs. Male	0.772 (0.308–1.937)	0.581	0.732 (0.292–1.838)	0.507
Coexisting disorders	Yes vs. No	1.154 (0.386–3.453)	0.797	0.692 (0.207–2.313)	0.550
Diabetes	Yes vs. No	0.706 (0.257–1.945)	0.380	0.622 (0.224–1.728)	0.363
Hypertension	Yes vs. No	1.122 (0.458–2.747)	0.801	0.960 (0.386–2.384)	0.929
Hyperlipidemia	Yes vs. No	0.677 (0.157–2.919)	0.601	0.729 (0.168–3.167)	0.673
Cardiovascular diseases	Yes vs. No	1.601 (0.638–4.015)	0.316	1.062 (0.380–2.970)	0.908
Cerebrovascular diseases	Yes vs. No	3.327 (0.975–11.356)	0.055	2.326 (0.612–8.848)	0.216
Cancer	Yes vs. No	0.536 (0.072–4.004)	0.543	0.410 (0.054–3.103)	0.388
Chronic renal diseases	Yes vs. No	3.678 (0.835–16.202)	0.085	3.437 (0.764–15.465)	0.108
Chronic liver diseases	Yes vs. No	1.433 (0.192–10.707)	0.726	0.997 (0.128–7.760)	0.997
Chronic Obstructive Pulmonary Disease	Yes vs. No	0.991 (0.230–4.272)	0.990	0.642 (0.139–2.955)	0.569
Neuropsychiatric disorders	Yes vs. No	1.186 (0.158–8.894)	0.868	0.734 (0.094–5.741)	0.768
History of surgery	Yes vs. No	1.168 (0.466–2.930)	0.740	1.041 (0.413–2.623)	0.932
Temperature at disease onset (°C)	37.4–39.0 vs. <37.4	1.514 (0.540–4.248)	0.430	1.844 (0.638–5.326)	0.258
	>39.0 vs. <37.4	1.036 (0.201–5.342)	0.966	2.714 (0.348–21.190)	0.622
Temperature at admission (°C)	37.4–39.0 vs. <37.4	1.476 (0.561–3.885)	0.430	1.721 (0.646–4.580)	0.277
	>39.0 vs. <37.4	2.630 (0.343–20.167)	0.352	2.714 (0.348–21.190)	0.341
Respiratory rate (/min)	≥20 vs. <20	1.357 (0.398–4.629)	0.626	1.316 (0.385–4.502)	0.662
Oxygen saturation (%)	≥90 vs. <90	0.131 (0.044–0.394)	<0.001	0.123 (0.041–0.369)	<0.001

* *Adjustments were made for age and sex*.

**Table 6:** Univariate and multivariate analyses of potential factors (laboratory indexes) predicting poor outcome.

**Factors**	**Normal range**	**Level^†^**	**Crude HR (95% CI)**	***P***	Adjusted HR (95% CI)^*^	***P***
**Hematologic**						
Leucocytes (×10^9^ /L)	3.5–9.5	≥9.5 vs. <9.5	4.634 (1.840–11.669)	0.001	5.575 (2.080–14.943)	0.001
Neutrophils (×10^9^ /L)	1.8–6.3	≥6.3 vs. <6.3	2.663 (1.102–6.433)	0.030	2.566 (1.022–6.443)	0.045
Lymphocytes (×10^9^ /L)	1.1–3.2	≥1.1 vs. <1.1	0.293 (0.068–1.266)	0.100	0.337 (0.077–1.475)	0.149
Monocytes (×10^9^ /L)	0.1–0.6	≥0.6 vs. <0.6	0.179 (0.024–1.335)	0.179	0.182 (0.024–1.366)	0.098
Platelets (×10^9^ /L)	125.0–350.0	≥125.0 vs. <125.0	0.733 (0.245–2.192)	0.578	0.837 (0.275–2.553)	0.755
Hemoglobin (g/L)	130.0–175.0	≥130.0 vs. <130.0	0.865 (0.354–2.116)	0.751	0.652 (0.244–1.732)	0.394
CD4+ T cells (%)	25.34–51.37	≥51.37 vs. <51.37	1.144 (0.439–2.980)	0.783	1.235 (0.468–3.259)	0.670
CD8+ T cells (%)	14.23–38.95	≥14.23 vs. <14.23	0.687 (0.249–1.890)	0.467	0.867 (0.303–2.482)	0.790
**Coagulation function**						
Prothrombin time (s)	11.0–16.0	≥13.5 vs. <13.5	1.574 (0.644–3.852)	0.320	1.112 (0.417–2.966)	0.832
Activated partial thromboplastin time (s)	28.0–43.5	≥43.5 vs. <43.5	1.363 (0.493–3.766)	0.551	1.204 (0.433–3.344)	0.722
Fibrinogen (g/L)	2.0–4.0	≥4.0 vs. <4.0	0.533 (0.212–1.338)	0.180	0.520 (0.205–1.317)	0.168
Thrombin time (s)	14.0–21.0	≥17.4 vs. <17.4	1.114 (0.463–2.678)	0.809	1.197 (0.489–2.930)	0.694
D-dimer (mg/L)	<0.5	≥0.5 vs. <0.5	1.232 (0.411–3.697)	0.710	0.940 (0.294–3.001)	0.917
**Biochemical**						
Alanine aminotransferase (U/L)	5–40	≥40 vs. <40	0.961 (0.393–2.351)	0.931	1.203 (0.429–3.373)	0.726
Aspartate aminotransferase (U/L)	8–40	≥40 vs. <40	1.611 (0.658–3.942)	0.296	1.900 (0.755–4.783)	0.173
Total bilirubin (μmol/L)	5.1–19.0	≥19.0 vs. <19.0	5.849 (2.433–14.063)	<0.001	6.171 (2.458–15.496)	<0.001
Total protein (g/L)	60–80	≥60 vs. <60	0.687 (0.284–1.661)	0.405	0.721 (0.298–1.748)	0.470
Albumin (g/L)	35–55	≥35 vs. <35	0.054 (0.007–0.405)	0.054	0.060 (0.008–0.460)	0.007
Globulin (g/L)	20–30	≥30 vs. <30	2.723 (1.113–6.666)	0.028	2.526 (1.027–6.211)	0.043
Prealbumin (mg/L)	170–420	≥170 vs. <170	1.001 (0.364–2.755)	0.999	1.282 (0.448–3.665)	0.643
Blood urea nitrogen (mmol/L)	2.9–8.2	≥8.2 vs. <8.2	6.283 (2.565–15.391)	<0.001	5.640 (2.193–14.509)	<0.001
Creatinine (μmol/L)	44–133	≥74 vs. <74	0.973 (0.405–2.337)	0.950	0.709 (0.254–1.978)	0.511
Creatine kinase (U/L)	38–174	≥174 vs. <174	2.039 (0.783–5.307)	0.144	1.982 (0.756–5.199)	0.164
Creatine kinase-MB (U/L)	0–24	≥24 vs. <24	2.449 (1.000–5.997)	0.050	3.032 (1.203–7.644)	0.019
Lactate dehydrogenase (U/L)	109–245	≥245 vs. <245	3.963 (0.915–17.161)	0.066	4.607 (1.057–20.090)	0.042
Hypersensitive cardiac troponin I (ng/L)	<26.2	≥26.2 vs. <26.2	5.613 (2.233–14.112)	<0.001	5.023 (1.921–13.136)	0.001
Glucose (mmol/L)	3.9–6.1	≥6.1 vs. <6.1	1.678 (0.637–4.416)	0.295	1.454 (0.543–3.893)	0.457
Serum potassium (mmol/L)	3.5–5.2	≥4.0 vs. <4.0	1.112 (0.462–2.677)	0.813	0.921 (0.368–2.304)	0.860
Serum sodium (mmol/L)	136–145	≥136 vs. <136	1.881 (0.436–8.120)	0.397	3.302 (0.702–15.535)	0.131
Serum calcium (mmol/L)	2.03–2.54	≥2.03 vs. <2.03	0.385 (0.160–0.925)	0.033	0.433 (0.173–1.083)	0.073
Serum phosphorus (mmol/L)	0.96–1.62	≥0.96 vs. <0.96	0.831 (0.345–2.001)	0.680	0.794 (0.330–1.914)	0.608
Serum chlorine (mmol/L)	96–108	≥96 vs. <96	1.617 (0.375–6.970)	0.519	2.195 (0.489–9.845)	0.305
Lactate concentration (mmol/L)	0.5–1.6	≥1.6 vs. <1.6	15.457 (2.067–115.615)	0.008	15.721 (2.099–117.777)	0.007
Positive Urinary protein	/	/	3.239 (1.244–8.433)	0.016	2.905 (1.099–7.678)	0.032
Positive Urinary glucose	/	/	0.893 (0.120–6.676)	0.912	0.961 (0.128–7.238)	0.969
Positive urinary occult blood	/	/	2.474 (1.030–5.945)	0.043	2.247 (0.932–5.421)	0.071
**Blood gas characteristics**						
pH	7.35–7.45	≥7.35 vs. <7.35	0.427 (0.170–1.074)	0.071	0.468 (0.181–1.207)	0.116
Arterial partial pressure of oxygen (mm Hg)	80–100	≥80 vs. <80	0.295 (0.098–0.883)	0.029	0.321 (0.106–0.973)	0.045
Arterial partial pressure of carbon dioxide (mm Hg)	35–45	≥45 vs. <45	2.159 (0.892–5.230)	0.088	2.224 (0.895–5.525)	0.085
**Infection-related biomarkers**						
Interleukin-2 (pg/mL)	0.1–4.1	≥4.1 vs. <4.1	1.820 (0.533–6.212)	0.339	2.343 (0.654–8.389)	0.191
Interleukin-4 (pg/mL)	0.1–3.2	≥3.2 vs. <3.2	1.663 (0.663–4.172)	0.279	2.112 (0.797–5.597)	0.133
Interleukin-6 (pg/mL)	0.1–2.9	≥23.3 vs. <23.3	1.782 (0.711–4.468)	0.218	1.485 (0.575–3.836)	0.414
Interleukin-10 (pg/mL)	0.1–5.0	≥5.0 vs. <5.0	2.629 (1.010–6.843)	0.048	3.551 (1.280–9.857)	0.015
Tumor necrosis factor-α (pg/mL)	0.1–23.0	≥2.6 vs. <2.6	1.079 (0.448–2.600)	0.866	1.032 (0.427–2.491)	0.945
Interferon-γ (pg/mL)	0.1–18.0	≥2.5 vs. <2.5	1.260 (0.522–3.043)	0.607	1.421 (0.582–3.473)	0.440
C-reactive protein (mg/L)	<8.0	≥65.0 vs. <65.0	4.703 (1.374–16.093)	0.014	5.275 (1.517–18.344)	0.009
Ferritin (ng/mL)	4.6–204.0	≥204.0 vs. <204.0	2.582 (0.210–11.930)	0.657	2.647 (0.311–22.522)	0.373

† *Adjustments were made for age and sex*.

* *Cut points of levels were determined according to normal range, actual distribution, and clinical significance*.

## Discussion

The global spread of SARS-CoV-2 poses a significant threat to public health. Previous studies have shown that 20% of COVID-19 patients developed critical disease due to hypoxia or respiratory failure. Among them, 5% require treatment in the ICU, while 15% require oxygen and essential care. This suggests that this is particularly important in understanding this part of the patient (10). Recently, Dong et al. found that children, particularly infants, developed severe outcomes (11). This indicated that patients of any age could develop severe illness. Feng et al. found that severe and critical patients with the typical characteristics of multiple organ and immune function dysfunction. They also found that older people aged ≥ 75 years are a risk factor for mortality (12). With the increase number of asymptomatic infectious patients, taking measures to detect and isolate early are especially important. In our study, we used a short-term method to prospectively study the reported the epidemiology and risk factors of 114 severe patients with COVID-19 from the Union hospital, Hubei province. To our knowledge, this is the first report to describes the severe patients with COVID-19 during a short-term observation and predict some risk factors for final outcome. In our study, the mean age of severe patients were 63.96 ± 13.41 years, and 58 (50.9%) were older than 65 years; the patients are thus older than in other studies (5, 13, 14). We also found that 78 (68.4%) of 114 patients initially exhibited fever, in accordance with previous studies, where fever is the one of the most common symptom in patients who had COVID-19 (5, 14–16). But, 36 (31.6%) of 114 severe patients did not exhibit fever at the beginning of illness, and other clinical manifestations should therefore be concerned. Recently, Jin et al. found that attention should also be paid to people who have gastrointestinal symptoms (6). Mao et al. indicated that clinicians should suspect COVID-19 also in patients with neurological manifestations (14).

According to results from laboratory tests, the poor outcome group had lower lymphocytes than the good outcome group [0.67 (0.43–0.89) vs. 0.92 (0.70–1.43)]. As is known to all, lymphocytes are the main fighting force against the virus, and we suspected that SARS-CoV-2 damages the lymphocyte and causes its reduction (17). Chen et al. found that severe lymphopenia were persistent and we more increased in dead patients than recovered patients, and they suggested that lymphopenia may be associated with poor outcome (18). Tan et al. demonstrated a contrasting result: lymphopenia is an effective indicator for the severity of patients with COVID-19 (19). CD8+ T cells were significantly lower in the poor outcome group. Chen et al. indicated that the SARS-CoV-2 infection may affect CD4+ and CD8+ T lymphocyte cells in particular and argue that this is a potential correlation with COVID-19 severity (20). In addition, markedly higher concentrations of cardiac troponin I, creatine kinase, and lactate dehydrogenase could be observed in the poor outcome group than in their counterpart. Most notably, patients who exhibited a poor outcome may develop pulmonary and extra-pulmonary organ damage, including septic shock, acute respiratory distress syndrome, acute kidney injury, acute cardiac injury, as well as disseminated intravascular coagulation. The fatality risk of COVID-19 patients with or without a history of previous cardiovascular disease may include acute cardiac injury and heart failure (18). Costanza Emanueli et al. suggested that the COVID-19 crisis will have long-term residual repercussions on the cardiovascular system (21). The suggestion is that the cardiac injury also requires special attention. In our study, we also found that lactate concentration was higher in poor outcomes than their counterpart. Lactate is generally the end product of energy through anaerobic metabolism, and the elevation of lactate levels is mainly caused by the increase of blood oxygen deficiency and anaerobic metabolism; this result is consistent with the lower oxygen saturation in the poor group, and this indicated that lactate level is an important predictor of poor outcome in the early stage. In addition, total bilirubin was also an important predictor of poor outcome in the early stage. Qi recommend that dynamic monitoring of the liver function of patients is necessary (22). Cai et al. conclude that patients with abnormal liver function may had higher risks of progressing to severe disease (23). Due to the “cytokine storm” also observed in the poor outcome group, 19 (95.0%) of these patients were given glucocorticoid therapy. Wu et al. previously found that the administration of methylprednisolone may have reduced the risk of death in patients with ARDS (7). To our surprise, most of the severe patients treated with Traditional Chinese medicine (TCM) were eventually converted to a good outcome, indicating the importance of this effort on COVID-19. A large of clinical practice results indicated that TCM shows significant role in the patients with COVID-19. For the severe patients in the treatment of TCM, the mean length in hospital and the time of nucleic acid turning negative has been shortened by more than 2 days (24). Yang et al. analyzed the effect of Lian Hua Qing Wen Capsules in the treatment of COVID-19 patients, and they found that this TCM could markedly relieve fever and cough and promote recovery (25). Besides, a comprehensive evaluation and further scientific research should be carried out on the effect of TCM on COVID-19.

Meantime, the risk factors related to the poor outcome included uncontrolled inflammation responses, infection, hypoxia, and liver, kidney, and cardiac dysfunction. The pathogenesis of COVID-19 is still being studied. Cytokine storms and uncontrolled inflammation responses are thought to play important roles in the outcome of COVID-19 (26–30). External stimuli resulted in an excessive immune response, and the pathogenesis of the cytokine storm is complex and can leaded to rapid disease progression and high mortality. The inflammatory cytokine storm is closely correlated to the development and progression of ARDS (31). Neutrophils play important role in chemokines and cytokines (32). In our study, the poor outcome group had significantly higher neutrophil counts than the good outcome group, and this may be the underlying cause of the cytokine storm. In addition, CD8^+^ T cells were significantly lower in the poor outcome group. These results highlight the important roles of CD8^+^ T cells in COVID-19. Studies had shown that T cells could inhibit the over-activation of innate immunity (33). T cells can help to clear SARS-CoV, and a low T-cell response can result in pathological changes in mice with SARS-CoV (34). The relevant mechanisms need to be studied further.

This study has some limitations. First, owing to the limited number of cases, only 114 severe patients were included. Second, this study was a single-center research, and a larger cohort study of severe patients with SARS-CoV-2 from other cities in China and other countries would help to further describe the clinical characteristics and predict risk factors related to this disease. Third, although we included numerous factors that may be associated with clinical outcome in the analyses and made adjustment for potential confounders when exploring the associations, we could not rule out the possibility of other residual confounders.

In summary, the present study is a single-center, prospective observational study that examined clinical characteristics and risk factors for poor short-term outcomes in patients with severe COVID-19. Our univariate and multivariate analyses demonstrated that cytokine storm/uncontrolled inflammatory responses as well as liver, kidney, and cardiac dysfunction may play important roles in determining final outcomes in patients with severe illness due to COVID-19 infection. Our data may aid clinicians in diagnosing severe cases of COVID-19 and determining the most appropriate treatment strategies for infected patients. Given that Traditional Chinese medicine has been shown to improve outcomes in some cases, additional studies are also required to assess the efficacy of such strategies.

## Data Availability Statement

The raw data supporting the conclusions of this article will be made available by the authors, without undue reservation.

## Ethics Statement

The studies involving human participants were reviewed and approved by the Ethics Commission of Wuhan Union Hospital of Tongji Medical College, Huazhong University of Science and Technology. Written informed consent was waived for the emergency of this infectious disease.

## Author Contributions

CY and WY designed the study, had full access to all data in the study, and took responsibility for the integrity of data and the accuracy of the data analysis. XF and LM contributed to data collection, literature search, and writing of the manuscript. XF and PL had roles in data analysis and data interpretation. All authors contributed to data acquisition and clinical management, and they reviewed and approved the final version of the manuscript.

## Conflict of Interest

The authors declare that the research was conducted in the absence of any commercial or financial relationships that could be construed as a potential conflict of interest.
